# Extracorporeal Life Support in a Porcine Model of Septic Endotoxemia with Acute Pulmonary Hypertension: An Experimental Study

**DOI:** 10.3390/jcm14176342

**Published:** 2025-09-08

**Authors:** Stany Sandrio, Joerg Krebs, Tobias Spanier, Grietje Beck, Manfred Thiel, Peter Tobias Graf

**Affiliations:** Department of Anesthesiology and Critical Care Medicine, University Medical Centre Mannheim, Medical Faculty Mannheim, University of Heidelberg, Theodor-Kutzer-Ufer 1-3, 68165 Mannheim, Germany; joerg.krebs@umm.de (J.K.); tspanier@t-online.de (T.S.); grietje.beck@umm.de (G.B.); manfred.thiel@medma.uni-heidelberg.de (M.T.); tobias.graf@umm.de (P.T.G.)

**Keywords:** sepsis, pulmonary hypertension, veno-arterial extracorporeal membrane oxygenation, veno-venoarterial extracorporeal membrane oxygenation, lipopolysaccharide, right ventricle

## Abstract

**Background**: This study evaluated the effects of veno-arterial (V-A) and veno-venoarterial (V-VA) ECMO in a porcine model of septic endotoxemia-induced acute pulmonary arterial hypertension (PAH). Our hypotheses were as follows: (1) V-VA ECMO lowers pulmonary vascular resistance (PVR) by delivering oxygenated blood to the pulmonary circulation, and (2) both V-A and V-VA ECMO improve perfusion to vital organs while simultaneously unloading the right ventricle (RV). **Methods**: Acute PAH was induced with *Salmonella abortus equi* lipopolysaccharide (LPS) in 34 pigs. Animals were randomized to either a control group without ECMO or to two groups receiving V-A or V-VA ECMO. **Results**: All animals developed PAH after one hour of LPS infusion: mean pulmonary artery pressure (PAP) increased significantly from 26 (24–30) mmHg to 40 (34–46) mmHg (*p* < 0.0001), and PVR increased from 314 (221–390) to 787 (549–1073) (*p* < 0.0001). Neither V-A nor V-VA ECMO significantly reduced PVR compared to controls. RV end-diastolic area increased in the control group [6.1 (4.3–8.6) cm vs. 8.5 (7.8–9.7) cm, *p* = 0.2], but not in the V-A [4.7 (3.3–7.6) cm] and V-VA [4.3 (2.5–8.3) cm] ECMO groups. Blood flow in the cranial mesenteric artery and celiac trunk did not differ significantly with or without ECMO. **Conclusions**: Elevating pulmonary artery oxygen tension through V-A or V-VA ECMO did not reduce PVR or PAP. However, both ECMO configurations effectively unloaded the RV and maintained perfusion to abdominal organs.

## 1. Introduction

Pulmonary arterial hypertension (PAH), defined as a mean pulmonary artery pressure (PAP) exceeding 20 mmHg at rest [1], has been observed in both experimental animal models [2,3] and critically ill patients with sepsis [2]. The resulting right ventricular (RV) dysfunction and dilation is multifactorial, caused by increased afterload due to pulmonary hypertension, the effects of positive pressure ventilation, and the direct impact of inflammatory cytokines on the myocardium [3]. It occurs in 30–60% of patients with sepsis and septic shock and is associated with more than a twofold increase in short- and long-term mortality [4] and is especially prevalent in patients with severe respiratory distress syndrome (ARDS) [5]. Notably, in SARS-CoV-2 pneumonia, acute PAH and RV dysfunction have also been reported in less advanced stages of disease, with prevalences of 12.0% and 14.5% among non-ICU patients, respectively [6]. In critically ill patients with SARS-CoV-2 infection, a dysregulated host response, involving hyperinflammation, coagulopathy, and pulmonary vascular dysfunction, further contributes to multiple organ failure [7].

In neonatal and pediatric patients, extracorporeal membrane oxygenation (ECMO) is recommended as a last-resort therapy for refractory septic shock [8]. In this setting, veno-arterial (V-A) ECMO may improve perfusion pressure and oxygen delivery to vital organs while simultaneously unloading the RV by reducing preload. This approach may significantly reduce catecholamine requirements, thereby limiting their adverse effects on the stressed myocardium. Additionally, veno-venoarterial (V-VA) ECMO may lower pulmonary vascular resistance by delivering oxygenated blood to the pulmonary circulation, thereby improving pulmonary hypertension and specifically addressing hypoxemia due to ARDS [9,10]. However, to date, the role of ECMO in general and the ideal cannulation modality in critically ill patients with acute pulmonary arterial hypertension remains uncertain.

This study aims to evaluate the cardiocirculatory effects of V-A and V-VA ECMO in a standardized porcine model of sepsis-induced acute pulmonary hypertension. We hypothesized that V-VA ECMO, compared to V-A ECMO, significantly reduces pulmonary artery pressure by delivering oxygen-rich blood to the pulmonary circulation. The primary endpoint was a reduction in mean PAP in the presence of a significant increase in pulmonary artery oxygen tension (PpaO_2_). Secondary endpoints included the ability of both ECMO modalities to maintain perfusion pressure to vital abdominal organs while simultaneously unloading the RV.

## 2. Materials and Methods

This study was approved by the Institutional Review Board for the care of animal subjects (Baden-Wuerttemberg Regional Council Karlsruhe, Department of Agriculture, Rural Areas, Veterinary and Food Affairs, Karlsruhe, Germany, 35-9185.81/G-9/22). All animals received humane care in compliance with the “Principles of Laboratory Animal Care” formulated by the National Society for Medical Research and the “Guide for the Care and Use of Laboratory Animals” prepared by the National Academy of Sciences, USA.

### 2.1. Animal Preparation

A total of 34 pigs (22 females, 12 males) weighing 25–35 kg (29.6 ± 2.6 kg) were fasted overnight with unrestricted access to water. Anesthesia was induced by i.m. injection of azaperone (120 mg), midazolam (45 mg), and ketamine (200 mg). The animals were then intubated with a cuffed 6.0 mm endotracheal tube and general anesthesia was maintained with 300 mg/h propofol (10 mg/kg/h) and 0.5 mg/h fentanyl (17 µg/kg/h) throughout the experiment. Animals were placed in the supine position and monitored via continuous ECG and pulse oximetry. Core temperature was maintained at 36–37 °C using a heating mat, stand heater, and pre-warmed infusions. The animals were mechanically ventilated in a volume-controlled mode with a positive end-expiratory pressure of 5 cm H_2_O, a tidal volume of 6 mL/kg, and a respiratory rate of 20–30 breaths per minute, adjusted to maintain a target PaCO_2_ of 35–45 mmHg. To control for potential confounders, the fraction of inspired oxygen, PEEP, and tidal volume were kept constant throughout the entire experiment. Mean arterial pressure was maintained at 60–70 mmHg; persistent hypotension was initially managed with a 300 mL bolus of pre-warmed balanced electrolyte solution. In case of volume refractory hypotension, norepinephrine was continuously infused.

All catheters and cannulas were inserted via direct puncture following atraumatic surgical exposure of the arteries and veins (for details see Appendix A) after administration of heparin (100 I.E./kg body weight). Correct catheter positions were verified through direct visualization at the end of the experiment.

The abdomen was accessed through a midline laparotomy extending from the xiphoid to approximately 4–5 cm above the symphysis. Urinary output was measured through bladder catheterization under direct visualization. The most distal parts of the inferior vena cava and abdominal aorta were exposed for ECMO cannulation. Further, the cranial mesenteric artery, celiac trunk, and infradiaphragmatic aorta were surgically exposed. To quantify arterial blood flow, appropriately sized flow probes (PS series, Transonic Systems Inc., Ithaca, NY, USA) were placed on these three vessels. To minimize fluid and heat loss, the abdominal wall was closed.

Thoracic access was gained via a median sternotomy. An appropriately sized flow probe (PAU series, Transonic Systems Inc., Ithaca, NY, USA) was placed on the pulmonary trunk to directly quantify cardiac output. To facilitate epicardial echocardiography, the sternal retractor was left in place, and the pericardial cavity was covered with surgical towels.

### 2.2. Experimental Design

**Figure 1:**
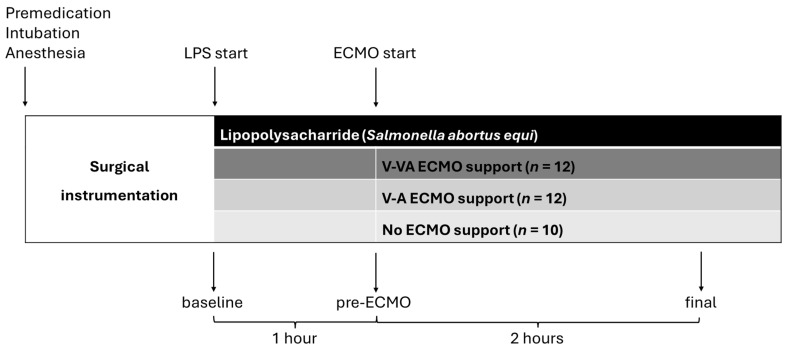
Experimental timeline. Hemodynamic measurements, echocardiography, and blood sampling were conducted at baseline, after one hour of continuous LPS infusion (pre-ECMO), and after a further two hours of LPS infusion with or without ECMO support (final). ECMO: extracorporeal membrane oxygenation; LPS: lipopolysaccharide; V-A: veno-arterial; V-VA veno-venoarterial.

Following instrumentation and baseline measurements, all animals received a continuous intravenous infusion of *Salmonella abortus equi* lipopolysaccharide (LPS) (L5886, Sigma-Aldrich, Taufkirchen, Germany) at a constant rate of 2.5 µg/kg/h. The experimental timeline is outlined in Figure 1.

The animals were randomly assigned to three groups: V-VA ECMO group (*n* = 12), V-A ECMO group (*n* = 12), and control group (*n* = 10) with either V-VA or V-A cannulation (5 per cannulation type) but no ECMO support. After one hour of continuous LPS infusion, ECMO support was initiated in the V-A and V-VA groups, based on the protocol established in our previous study [11].

As one of the aims of our study was to assess the ability of both ECMO modalities to maintain perfusion pressure to vital abdominal organs, V-A ECMO flow rate was set to 50% of baseline cardiac output. With preserved LV function, arterial support at ~50% of cardiac output was sufficient to sustain a watershed point between the thoracic and abdominal aorta, as demonstrated in our pilot study. In the V-VA configuration, an additional flow of ~1 L/min (30–40% of cardiac output) was returned through the jugular cannula, which was estimated to provide adequate oxygen delivery to the upper body circulation.

ECMO support was limited to 2 h, as this duration was considered sufficient to demonstrate a significant reduction in PAP and PVR. According to the literature, significant reductions in PAP and PVR can be expected within minutes of improved pulmonary artery oxygen tension [12,13]. In both ECMO groups, the final evaluation was performed under ongoing ECMO support.

Extracorporeal gas flow was kept constant at a rate of 0.5 L/min with 100% oxygen throughout the experiment. Arterial (SaO_2_) and mixed venous (SvO_2_) blood samples were collected at baseline, one hour after the initiation of LPS infusion (pre-ECMO), and two hours thereafter (final). Samples were drawn into heparinized blood gas syringes and immediately analyzed, with all values corrected for core body temperature (Siemens RAPIDPoint 500, Siemens Healthineers AG, Forchheim, Germany). Pulmonary artery oxygen tension (PpaO_2_) was measured from pulmonary artery blood samples in all groups. Mixed venous blood (SvO_2_) samples were collected from the pulmonary artery in the control group, whereas in the V-A and V-VA ECMO groups, samples were obtained from the pre-oxygenator site. Systemic arterial, left ventricular, pulmonary arterial, and central venous pressures were continuously monitored. Pulmonary capillary wedge pressure was obtained intermittently.

Using cardiac output (*CO*) measured at the pulmonary trunk, pulmonary and systemic vascular resistance (*PVR* and *SVR*) were then calculated as follows:

$$PVR=80\;\times \frac{\left(mPAP-PCWP\right)}{CO}$$

$$SVR=80\;\times \frac{\left(MAP-CVP\right)}{CO}$$

where *mPAP* is mean pulmonary artery pressure, *PCWP* is pulmonary capillary wedge pressure, and *MAP* is mean arterial pressure; *CVP* is central venous pressure.

The heart was imaged using parasternal and apical-equivalent echocardiographic planes. Direct epicardial echocardiography was performed by placing a standard echocardiographic transducer, enclosed in a sterile sheath, directly on the epicardial surface. Two-dimensional and Doppler echocardiographic assessments were conducted to evaluate basal end-diastolic diameters of both ventricles, septal bowing or paradoxical septal motion, significant valvular regurgitation or stenosis, and intracardiac shunts. RV function was assessed using tricuspid annular plane systolic excursion (TAPSE) and RV fractional area change, while left ventricular (LV) function was evaluated using Doppler mitral inflow, tissue Doppler imaging of the mitral annulus, and LV velocity–time integral. Due to significant LV shortening, ejection fraction could not be measured reliably.

### 2.3. Statistical Analysis

Based on data from the LPS model used in our previous study [11], an estimated sample size of twelve animals was required to measure differences in pulmonary artery pressure between ECMO groups with a power of 0.8 and a significance level of 0.05. Results are presented as mean ± standard deviation for normally distributed values and as median with interquartile range for values without a normal distribution. The within-subject effect of time (differences between baseline and pre-ECMO) was assessed using analysis of variance (F-test) with correction for repeated measurements. As various values at final evaluation were not normally distributed, between-group differences were analyzed using the non-parametric Steel–Dwass method for multiple comparisons. Data were analyzed using JMP Version 16 (JMP Statistical Discovery LLC., Cary, NC, USA).

## 3. Results

There were no significant differences in relevant physiological parameters at baseline and at pre-ECMO (after 1 h of LPS infusion) between the V-VA ECMO, V-A ECMO, and control groups.

**Figure 2:**
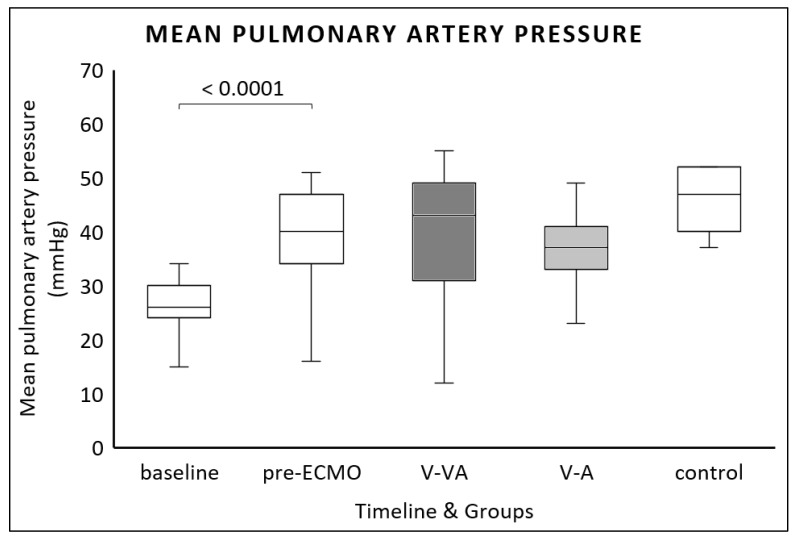
Mean pulmonary artery pressure (PAP) measured at baseline, after one hour of continuous LPS infusion (pre-ECMO), and at the final evaluation in the V-VA ECMO group (dark gray), the V-A ECMO group (light gray), and the control group without ECMO support (white).

All animals developed PAH at pre-ECMO. Mean PAP increased significantly from baseline to pre-ECMO (26 (24–30) mmHg vs. 40 (34–46) mmHg, *p* < 0.0001). We found no differences between the two ECMO modalities (Figure 2).

**Figure 3:**
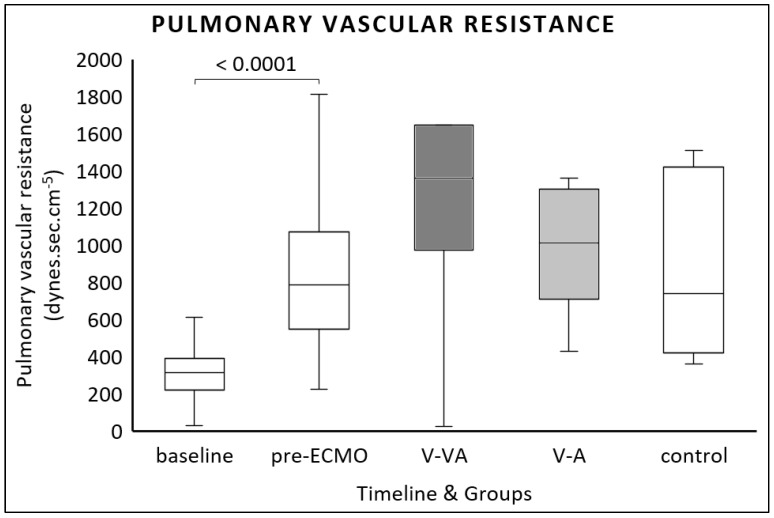
Pulmonary vascular resistance (PVR) calculated at baseline, after one hour of continuous LPS infusion (pre-ECMO), and at the final evaluation in the V-VA ECMO group (dark gray), the V-A ECMO group (light gray), and the control group without ECMO support (white).

PVR increased from baseline to pre-ECMO (314 (221–390) dynes.s.cm^−5^ vs. 787 (549–1073) dynes.s.cm^−5^, *p* < 0.0001). Compared to the control group, neither V-A nor V-VA ECMO reduced PVR at the final evaluation (Figure 3). Furthermore, we found no differences between the ECMO modalities.

RV end-diastolic area remained stable from baseline to pre-ECMO (6.1 (4.3–8.6) cm vs. 5.8 (4.5–8.1) cm, *p* = 0.7). Both ECMO strategies were equally effective in limiting RV distension compared to control at the final evaluation (4.7 (3.3–7.6) vs. 8.5 (7.8–9.7), *p* = 0.02 for V-A ECMO vs. control, respectively; and 4.3 (2.5–8.3) vs. 8.5 (7.8–9.7), *p* = 0.04 for V-VA ECMO vs. control) (Table 1).

From baseline to pre-ECMO, SvO_2_ decreased (75 (72–80)% vs. 71 (63–74)%, *p* = 0.0003). At the end of the experiment, SvO_2_ was significantly lower in the control group compared to both ECMO strategies (V-A ECMO, 72 (65–81)% vs. 54 (38–68)%, *p* = 0.002; V-VA ECMO, 70 (67–82)% vs. 54 (38–68)%, *p* = 0.002).

**Figure 4:**
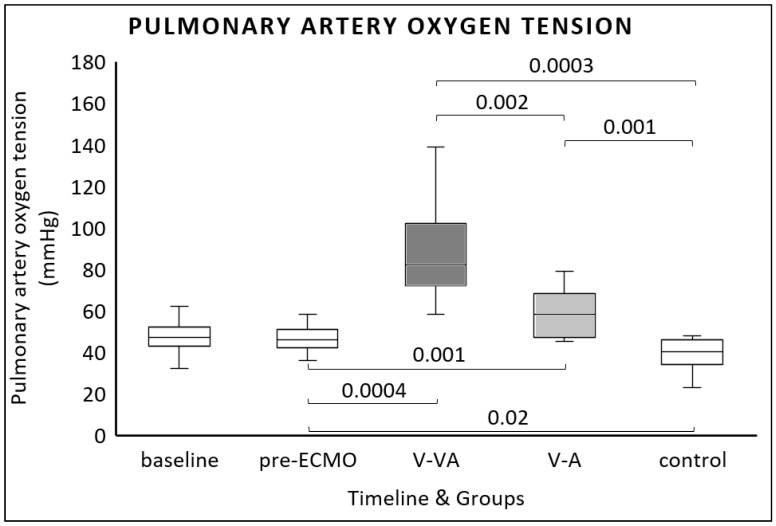
Pulmonary artery oxygen tension (PpaO_2_) measured at baseline, after one hour of continuous LPS infusion (pre-ECMO), and at the final evaluation in the V-VA ECMO group (dark gray), the V-A ECMO group (light gray), and the control group without ECMO support (white).

At the end of the experiment, both ECMO modalities significantly increased pulmonary arterial oxygen tension (PpaO_2_) compared to pre-ECMO values [46 mmHg (42–50 mmHg) vs. 58 mmHg (47–68 mmHg) for V-A ECMO; *p* = 0.001 and 82 mmHg (72–102 mmHg) for V-VA ECMO; *p* = 0.0004] (Figure 4). Additionally, V-VA ECMO resulted in significantly higher PpaO_2_ at the end of the study compared to V-A ECMO [82 mmHg (72–102 mmHg) vs. 58 mmHg (47–68 mmHg); *p* = 0.002] (Figure 4).

Arterial oxygen tension (PaO_2_) decreased from baseline to pre-ECMO (220 mmHg (206–230 mmHg) vs. 195 mmHg (147–223 mmHg), *p* = 0.0001) (Table 2). In the control group, PaO_2_ declined further at the end of the experiment (99 mmHg (74–127 mmHg) vs. 208 (146–242) and 190 (148–246) for V-VA and V-A ECMO, respectively, *p* = 0.002 and *p* = 0.02) (Table 2).

Blood flow in the cranial mesenteric artery remained stable throughout the experiment (Table 3). At the celiac trunk, blood flow increased from baseline to pre-ECMO (0.15 L/min (0.1–0.3) vs. 0.4 (0.1–0.6, *p* = 0.0001). Thereafter, no significant changes in blood flow were observed (Table 3).

From baseline to pre-ECMO, blood flow in the infradiaphragmatic aorta decreased (1.8 L/min (1.3–2.9 L/min) vs. 1.7 L/min (1.1–2.1 L/min), *p* = 0.03). V-A and V-VA ECMO induced comparable retrograde flow in the infradiaphragmatic aorta (Table 3).

## 4. Discussion

In this study, we evaluated the cardiocirculatory effects of veno-arterial (V-A) and veno-venoarterial (V-VA) ECMO cannulation strategies in a standardized porcine model of LPS-induced acute PAH. Our main findings demonstrated that (1) neither V-VA nor V-A ECMO effectively reduced PVR or PAP; and (2) both ECMO modalities maintained abdominal blood flow while effectively unloading the RV.

In ARDS, PVR is typically increased by low PpaO_2_, hypercapnia, acidosis, and high airway pressures [14]. On the other hand, endotoxin inhibits hypoxic pulmonary vasoconstriction, leading to a right-to-left intrapulmonary shunt and systemic hypoxia [14]. The primary advantage of V-VA ECMO over V-A ECMO is its ability to prevent low PpaO_2_ and differential hypoxia if cardiac function recovers before pulmonary function, thereby protecting critical organs such as the heart and brain [15,16].

Our results indicate that increasing PpaO_2_ via ECMO while maintaining normocapnia did not reduce the LPS-induced increase in PVR. However, this approach prevented a significant decline in PaO_2_ associated with right-to-left intrapulmonary shunting, as observed in the control group.

Experimental lung injury models with atelectatic lungs have shown that increasing PpaO_2_ above 100 mmHg can effectively reduce PVR [12]. Accordingly, some authors propose that V-V ECMO might lower PVR by increasing PpaO_2_ during severe ARDS, thereby reducing RV afterload and improving RV function [17,18]. This effect could be mediated by improved PpaO_2_, increased CO_2_ elimination, correction of respiratory acidosis, and reduced airway pressures [19,20]. In a porcine model of endotoxin shock, Mu et al. reported a return of PVR to baseline levels under V-A ECMO support [21]. However, in this study, a lung-rest strategy was employed, including a reduced respiratory rate, making it unclear whether the observed PVR reduction was attributable to increased PpaO_2_ or to changes in ventilator settings. In contrast, Holzgraefe et al. found that increasing PpaO_2_ via peripheral V-V ECMO, without altering ventilator settings, failed to reduce mean pulmonary artery pressure or lessen hypoxic pulmonary vasoconstriction in a porcine model of global alveolar hypoxia [16]. Thus, based on the existing literature and our findings, increasing PpaO_2_ via peripheral V-VA ECMO ensures adequate oxygenation but does not necessarily decrease PVR.

Sudden, sustained elevations in PVR may compromise RV contractility, leading to rapid RV distension and failure [22]. RV failure has been reported in 22.5% of patients with septic shock [23] and in 25% of ARDS patients, a condition linked to decreased survival in those requiring V-V ECMO [22,24]. In our study, both V-A and V-VA ECMO facilitated RV unloading through venous-arterial shunting. After two hours of ECMO support, epicardial echocardiography showed a significantly smaller RV end-diastolic diameter in both ECMO groups compared to the control group, but no difference between V-A and V-VA ECMO.

Both V-A and V-VA ECMO strategies effectively maintained perfusion pressure to vital abdominal organs and allowed partial weaning from catecholamines [25,26], thereby reducing their adverse effects on the myocardium, PVR, and overall organ function [25,27,28]. Mixed venous oxygen saturation (SvO_2_), a surrogate marker for assessing the adequacy of systemic oxygen delivery, declined in the control group to levels that, from a clinical perspective, would be considered inadequate in critically ill patients [29]. In contrast, SvO_2_ was significantly higher in both the V-VA and V-A ECMO groups, indicating that both ECMO modalities effectively maintained global oxygen delivery.

However, these findings should be interpreted with caution, as SvO_2_ was measured from the pre-oxygenator site in both ECMO groups [15]. In the V-VA configuration, arterialized blood is partially returned to the venous circulation, potentially leading to an overestimation of true SvO_2_ values if measured directly from pulmonary artery blood samples. In V-A ECMO, blood is primarily drained from the inferior vena cava, while venous return from the superior vena cava largely bypasses the drainage cannula [15]. Since the primary focus of this study was on perfusion and oxygen delivery to abdominal organs, SvO_2_ was specifically measured at the pre-oxygenator site.

The adequacy of abdominal perfusion could also be confirmed by sustained blood flow in the cranial mesenteric artery and celiac trunk, with no statistical differences between the study groups. The slight negative blood flow observed at the proximal abdominal aorta in both ECMO groups suggests that, with arterial ECMO flow set at 50% of baseline cardiac output and preserved left ventricular function, the mixing point between native and retrograde ECMO circulations (the watershed region) is located between the thoracic and abdominal aorta.

Using continuous intravenous infusion of *Salmonella abortus equi* LPS, we established a reproducible model of acute precapillary PAH. After one hour, mean PAP consistently exceeded 30 mmHg, confirming this timeframe as sufficient to reach the experimental target. The pulmonary hypertension was precapillary in nature, driven by endotoxin-induced pulmonary vasoconstriction and reflected by elevated mean PAP and PVR, with low pulmonary capillary wedge pressure and LVEDP. During V-A ECMO, additional venous return through a jugular cannula raises pulmonary artery oxygen tension and should theoretically reduce PAP and PVR in precapillary PAH [12]. However, our study failed to demonstrate such an effect, as discussed previously. This mechanism must be clearly differentiated from LV venting with an additional ECMO drainage cannula [30,31], which addresses postcapillary PAH and cardiogenic pulmonary edema. Postcapillary PAH typically arises from cardiogenic shock and left-sided heart disease, characterized by elevated LVEDP and pulmonary venous pressure [32]. While LV venting reduces LVEDP and prevents pulmonary congestion [30,33], it does not target the underlying pathophysiology of precapillary PAH.

### Limitations

Our study is an experimental investigation using a porcine model, and its findings should be cautiously translated into clinical practice. We used a continuous LPS infusion to induce an increase in PVR. This represents an artificial model, mimicking only aspects of septic shock and extrapulmonary ARDS [34]. Further, LPS also inhibits hypoxic pulmonary vasoconstriction, leading to a right-to-left intrapulmonary shunt and systemic hypoxia [14], which might have influenced our findings.

Changes in PVR and PAP are dynamic and often occur over a longer period of time [35]. In our unpublished pilot study, however, one hour of LPS infusion consistently increased mean PAP to values above 30 mmHg, confirming this timeframe as sufficient to establish acute PAH. Accordingly, we investigated a reproducible model of acute PAH, defined by a significant rise in mean PAP compared with baseline [11]. Short-term V-V ECMO support, however, may not be sufficient to achieve a clinically relevant reduction in PAP [36]. In contrast, Reis et al. reported an immediate decrease in PAP following V-V ECMO initiation [18]. Thus, time-dependent effects of ECMO on PAP and PVR might be influenced by the ECMO modality and the underlying disease.

To minimize the influence of ventilator settings and alveolar oxygen tension on PVR, ventilatory parameters were maintained without transitioning to ultraprotective ventilator settings during ECMO support. Traditional ventilator strategies are known to influence respiratory system function and hemodynamics both in experimental ARDS [37] as well as in clinical practice [19,20]. Drugs with alpha- and beta-agonistic effects influence pulmonary vascular tone and consequently PVR [38]. Therefore, we tried to avoid the administration of norepinephrine during our study. Lastly, epicardial echocardiographic assessments in this study cannot be directly compared with standardized transthoracic or transesophageal echocardiography used in clinical practice.

## 5. Conclusions

In a porcine model with LPS-induced acute PAH, increasing pulmonary artery oxygen tension through either V-A or V-VA ECMO did not reduce PVR or PAP. Nevertheless, both ECMO configurations effectively unloaded the RV and maintained adequate perfusion to vital abdominal organs.

## Figures and Tables

**Table 1:** Physiological parameters.

	Baseline	Pre-ECMO	Final V-VA Group	Final V-A Group	Final Control Group	V-A vs. V-VA	V-A vs. Control	V-VA vs. Control
	*n* = 34	*n* = 34	*n* = 12	*n* = 12	*n* = 10			
ECMO total flow [L/min]			2.3 (2.2–2.7)	1.7 (1.5–2.2)		**0.04**		
ECMO arterial flow [L/min]			1.3 (1.2–1.7)	1.7 (1.5–2.2)		**0.01**		
Mean arterial pressure [mmHg]	82 (73–99)	100 (88–113)	78 (61–102)	75 (67–88)	97 (85–102)	0.9	0.2	0.6
Norepinephrine [µg/kg/min]	0.1 (0.01–0.3)	0.01 (0.01–0.1)	0.1 (0.01–1.8)	0.1 (0.02–5.2)	0.01 (0.01–0.01)	0.8	**0.02**	0.1
Fluid substitution [L]	6.3 (4.3–7.3)		8 (6–10.7)	7.4 (6.2–9.6)	4.5 (4–7.7)	0.9	0.1	**0.02**
Heart rate [beat/min]	85 (74–110)	130 (95–152)	129 (89–148)	120 (102–173)	101 (64–144)	0.9	0.4	0.7
Pulmonary circulation								
Central venous pressure [mmHg]	14 (13–15)	14 (13–15)	15 (11–16)	15 (13–17.5)	17 (16–20)	0.6	0.1	**0.02**
Systolic PAP [mmHg]	34 (30–38)	53 (42–60)	60 (39–69)	47 (43–51)	56 (52–70)	0.2	**0.04**	1
Diastolic PAP [mmHg]	21 (19–24)	31 (25–37)	33 (29–39)	29 (25–34)	37 (31–39)	0.5	0.3	0.9
Mean PAP [mmHg]	26 (24–30)	40 (34–46)	42 (30–48)	36 (33–41)	47 (39–52)	0.5	0.07	0.7
Transonic cardiac output [L/min]	2.7 (2.1–3.3)	2.6 (1.9–3)	1.7 (0.9–2.3)	1.7 (1–1.9)	3 (1.9–3.9)	1	**0.05**	0.07
RV diameter [cm]	2.5 (2.2–2.9)	2.3 (1.9–2.5)	2.1 (1.6–2.6)	2.2 (1.8–2.6)	2.8 (2.6–3)	1	**0.02**	**0.02**
RV end-diastolic area [cm^2^]	6.1 (4.3–8.6)	5.8 (4.5–8.1)	4.3 (2.5–8.3)	4.7 (3.3–7.6)	8.5 (7.8–9.7)	0.9	**0.02**	**0.04**
Systemic circulation								
PCWP [mmHg]	16 (14–17)	16 (14.5–17)	15 (14–20)	16 (14–19)	18 (16–20)	0.9	0.7	0.5
LVEDP [mmHg]	14 (12–15)	13 (11–15)	12 (6.5–14.7)	13 (9–15)	14.5 (13–17.5)	0.7	0.5	0.2
LVESP [mmHg]	109 (100–123)	130 (107–146)	116 (110–139)	112 (101–126)	117 (107–120)	0.6	1	0.9
LV diameter [cm]	3.5 (3–3.8)	3.1 (2.8–3.6)	2.9 (2.5–3.4)	3 (2.6–3.1)	3.6 (3.2–3.8)	1	**0.01**	0.07
E/E’	5.8 (4.2–7.4)	5.9 (3.9–10)	5.3 (4.3–6.3)	7.1 (3.6–10.9)	7.4 (4.1–12.1)	0.7	0.9	0.5
S’	7.5 (6.8–10.5)	7.8 (6–10)	6.4 (5.8–10.8)	6.6 (6.1–8.3)	8 (7.3–10.5)	1	0.1	0.3
SVR [dynes.s.cm^−5^]	2167 (1728–2637)	2821 (2162–3528)	3294 (1625–6022)	2449 (1803–3728)	1924 (1402–2821)	0.7	0.6	0.3
Mechanical ventilation								
Respiratory rate [breath/min]	26 (20–28)	28 (22–28)	28 (13–29)	26 (16–28)	24 (20–26)	1	0.9	0.7
Plateau pressure [mbar]	11 (10–12)	13 (12–15)	18 (15–20)	18 (15–21)	17 (15–20)	0.9	0.9	1
PEEP [mbar]	5	5	5	5	5	1	1	1
Tidal volume [mL]	184 (178–194)	186 (173–200)	188 (175–196)	189 (181–203)	179 (174–208)	1	0.7	0.9

Hemodynamic measurements at baseline, after one hour of continuous lipopolysaccharide infusion (pre-ECMO), and at final evaluation without (control) or with ECMO support (V-A and V-VA). Results are presented as median and interquartile range (Q1/Q3). Between-group differences at final evaluation were analyzed using the non-parametric Steel–Dwass method for multiple comparisons. PAP: pulmonary artery pressure; RV: right ventricle; PCWP: pulmonary capillary wedge pressure; LVEDP: left ventricular end-diastolic pressure; LVESP: left ventricular end-systolic pressure; LV: left ventricle; SVR: systemic vascular resistance; PEEP: positive end-expiratory pressure. Background shading is applied to distinguish subsections within the table. Bold = statistically significant.

**Table 2:** Blood gas analyses.

	Baseline	Pre-ECMO	Final V-VA Group	Final V-A Group	Final Control Group	V-A vs. V-VA	V-A vs. Control	V-VA vs. Control
	*n* = 34	*n* = 34	*n* = 12	*n* = 12	*n* = 10			
Arterial pH	7.42 (7.38–7.45)	7.38 (7.35–7.41)	7.42 (7.31–7.45)	7.40 (7.28–7.47)	7.36 (7.29–7.38)	1	0.7	0.4
PaO_2_ [ mmHg]	220 (206–230)	195 (147–223)	208 (146–242)	190 (148–246)	99 (74–127)	1	**0.02**	**0.002**
SaO_2_ [%]	99.7 (99.6–99.7)	99.7 (98.9–99.7)	99 (98–99)	99 (98–100)	96 (85–98)	0.9	0.05	**0.02**

PaO_2_: arterial oxygen tension; SaO_2_: arterial oxygen saturation; ECMO: extracorporeal membrane oxygenation; V-A: veno-arterial; V-VA: veno-venoarterial. Bold = statistically significant.

**Table 3:** Abdominal perfusion.

	Baseline	Pre-ECMO	Final V-VA Group	Final V-A Group	Final Control Group	V-A vs. V-VA	V-A vs. Control	V-VA vs. Control
	*n* = 34	*n* = 34	*n* = 12	*n* = 12	*n* = 10			
Transonic celiac trunk [L/min]	0.15 (0.1–0.3)	0.4 (0.1–0.6)	0.2 (0.1–0.4)	0.3 (0.1–0.5)	0.2 (0.1–0.3)	0.7	0.4	0.9
Transonic CMA [L/min]	0.6 (0.5–0.8)	0.5 (0.3–0.7)	0.7 (0.5–0.9)	0.7 (0.6–1.3)	0.6 (0.5–0.8)	0.8	0.7	1
Transonic abdominal aorta [L/min]	1.8 (1.3–2.9)	1.7 (1.1–2.1)	−0.1 (−0.2–0.4)	−0.2 (−0.6–0)	1.2 (0.9–2)	0.4	**0.0004**	**0.002**

Abdominal perfusion at baseline, after one hour of continuous lipopolysaccharide infusion (pre-ECMO), and at final evaluation without (control) or with ECMO support (V-A and V-VA). Results are presented as median and interquartile range (Q1/Q3). Between-group differences at final evaluation were analyzed using the non-parametric Steel–Dwass method for multiple comparisons. CMA: cranial mesenteric artery; ECMO: extracorporeal membrane oxygenation; V-A: veno-arterial; V-VA: veno-venoarterial. Bold = statistically significant.

## Data Availability

The datasets analyzed for this study are available from the corresponding author upon reasonable request.

## Abbreviations

The following abbreviations are used in this manuscript:

CMA	Cranial mesenteric artery
ECMO	Extracorporeal membrane oxygenation
LV	Left ventricle
LVEDP	Left ventricular end-diastolic pressure
LVESP	Left ventricular end-systolic pressure
PAP	Pulmonary artery pressure
PAH	Pulmonary arterial hypertension
PaO_2_	Arterial oxygen tension
PpaO_2_	Pulmonary arterial oxygen tension
PCWP	Pulmonary capillary wedge pressure
PEEP	Positive end-expiratory pressure
PVR	Pulmonary vascular resistance
RV	Right ventricle
SaO_2_	Arterial oxygen saturation
SvO_2_	Mixed venous oxygen saturation
SVR	Systemic vascular resistance
V-A	Veno-arterial
V-VA	Veno-venoarterial

## Appendix A

Vascular access sites and utilization:

1. 20G peripheral intravenous line in the left auricular vein:
  - Induction of anesthesia, fluid administration.
2. Central venous catheter in the left internal jugular vein:
  - Continuous infusion of anesthetic drugs, norepinephrine, lipopolysaccharide, and fluids;
  - Continuous measurement of central venous pressure;
  - Transpulmonary thermodilution.
3. Pulmonary artery catheter in the right internal jugular vein:
  - Continuous pulmonary artery pressure measurement, pulmonary capillary wedge pressure measurement;
  - Pulmonary artery thermodilution measurement;
  - Central venous and mixed venous blood gas analysis.
4. Arterial catheter in the left carotid artery:
  - Hemodynamic monitoring;
  - Arterial blood gas analysis.
5. Left ventricular catheter via the right carotid artery:
  - Continuous left ventricular pressure monitoring.
6. Venous ECMO drainage cannula in the inferior vena cava.
7. Venous ECMO return cannula in the left brachiocephalic vein.
8. Arterial ECMO return cannula in the distal abdominal aorta.
